# Evaluation of the Clinical Outcomes Associated With the Use of Fatty Acids and Vitamin D in Rheumatoid Arthritis Patients: A Systematic Review and Meta‐Analysis

**DOI:** 10.1002/fsn3.70473

**Published:** 2025-07-21

**Authors:** Bing Xu, Dongdong Liang, Guangfeng Chen

**Affiliations:** ^1^ Department of Rheumatology Affiliated Hospital of Shandong University of Traditional Chinese Medicine Jinan China; ^2^ Department of Life Science , Jinan Health Care Development Center Jinan Shandong China; ^3^ Department of Geriatric Medicine Affiliated Hospital of Shandong University of Traditional Chinese Medicine Jinan China

**Keywords:** fatty acids, omega‐3 fatty acids, rheumatoid arthritis, vitamin D

## Abstract

Non‐pharmacological therapies such as dietary interventions, fatty acids and vitamin supplementation, and physical changes are emerging as complementary treatments in the management of rheumatoid arthritis. Fatty acids (omega‐3 fatty acids) and vitamin D modulate inflammatory pathways and decrease the production of inflammatory mediators having a beneficial effect on RA symptoms. The meta‐analysis synthesized data from randomized controlled trials (RCTs) and investigated the effects of fatty acids and vitamin D supplementation in RA patients using clinical outcomes. A systematic literature review was conducted using MEDLINE and Central databases for RCTs that involved the administration of fatty acids (*n* = 14 trials) or vitamin D (*n* = 10 trials) versus conventional treatment adult RA patients. Clinical outcomes such as Disease Activity Score in 28 joints (DAS28), tender and swollen joint counts (TJC and SJC), patients global assessment (PGA), health assessment questionnaire (HAQ) and the visual analogue scale (VAS) for pain assessment were determined at baseline and the end of the study. Meta‐analysis was performed using RevMan to assess the risk of bias and determine the mean differences and significance between the intervention and control groups. Data were analyzed from 24 RCTs with 1713 patients. Fatty acid administration caused a significant improvement in DAS28 (*p* < 0.0001), TJC (*p* < 0.0001) and HAQ scores (*p* < 0.00001) but there was no significant difference in SJC, PGA, and VAS scores relative to controls. In contrast, vitamin D supplementation only significantly improved HAQ (*p* = 0.02). However, fatty acid and vitamins showed a favorable effect on most clinical outcomes, which may be clinically relevant. This work suggests a moderate improvement in certain domains of clinical outcomes of RA patients with fatty acids and vitamin D. Since the trials used in the analysis were heterogenous and had a limited sample size, larger, multi‐centre trials are needed to validate the efficacy of these interventions.

## Graphical abstract

Non‐pharmacological therapies, including dietary interventions, fatty acids, and vitamin supplementation, as well as physical modifications, are gaining recognition as complementary approaches in the management of rheumatoid arthritis. Omega‐3 fatty acids and vitamin D play a significant role in modulating inflammatory pathways and reducing the production of inflammatory mediators, thereby exerting a positive influence on the symptoms of rheumatoid arthritis. The meta‐analysis integrated findings from randomized controlled trials (RCTs) and examined the impact of fatty acid and vitamin D supplementation on clinical outcomes in patients with rheumatoid arthritis (RA). This meta‐analysis shows that fatty acids and vitamin D can reduce pain, improve quality of life, and reduce arthritic symptoms. Fatty acids enhance inflammation outcomes such joint soreness and edema and increase patient function. Outcome measures showed moderate vitamin D supplementation effectiveness.

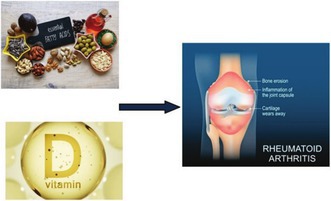

## 1 Introduction

Rheumatoid arthritis (RA) is a chronic autoimmune disease that causes inflammation of the joints, resulting in symptoms such as pain, tenderness, stiffness, swelling, and loss of function in joints (National Institute of Arthritis and Musculoskeletal and Skin Diseases, n.d.). Progressive joint damage caused by RA can eventually result in bone erosion and joint deformity and result in problems to the heart, lungs, and nervous system (World Health Organization, n.d.; NHS, n.d.). Globally, RA affects 0.24%–1% of the adult population and is two‐ to three‐fold more prevalent in women than men (Suresh et al. 2024; Venetsanopoulou et al. 2023). In addition to the direct effects on the health of patients, RA can pose a significant psychological and economic burden on societies and healthcare systems. Joint deformities result in higher disability rates in RA patients, causing lower quality of life, and direct costs caused by drug treatments, work disability, and productivity losses can drain healthcare systems. The average annual cost of RA patients was estimated to be between $12,500 and $36,000 annually and was mainly associated with drug costs including biologicals (Huang et al. 2024).

Although the exact pathophysiology of RA has not been elucidated, dysregulated inflammatory processes have been implicated in the development of RA and involve an interplay between genetic factors, environmental triggers (smoking, infections, gut dysbiosis), and immune responses (production of auto antibodies) (Gao et al. 2024). The management of RA involves the use non‐steroidal anti‐inflammatory drugs (NSAIDs) such as ibuprofen, meloxicam, and naproxen and corticosteroids that are used to relieve pain and inflammation but are non‐specific in action and can lead to adverse effects following extended use. Disease‐modifying antirheumatic drugs (DMARDs) such as methotrexate, salufasalazine, hydroxychloroquine, and leflunomide are the cornerstone of RA treatment act by blocking the effect of inflammatory mediators and preventing joint destruction whereas biological DMARDs (infliximab, etanercept, tocilizumab, etc.) also block inflammatory responses but in a selective and targeted manner (Gao et al. 2024; Benjamin et al. 2025). The high cost and side effects associated with DMARDs has led to several non‐pharmacological treatments such as vitamin supplementation, dietary modifications, fatty acids, and physical training being explored for the management and supportive care of patients with RA (Kostoglou‐Athanassiou et al. 2020, 2012).

Omega‐3 fatty acids modulate the inflammatory response by acting as precursors to lipid mediators of inflammation and have been shown to decrease the production of interleukins (IL‐1β and IL‐6), tumor necrosis factor (TNF‐α), and interferons (INF‐γ) in in vitro, animal, and human models, thus reducing the inflammatory response in RA (Kostoglou‐Athanassiou et al. 2020). A cohort study showed a high prevalence of vitamin D deficiency in RA patients and a link to disease severity, and vitamin D has been shown to control innate and adaptive immune responses by inhibiting cytokine levels. Based on this information, fatty acids and vitamin D supplementation are expected to improve symptoms and clinical responses in RA patients (Kostoglou‐Athanassiou et al. 2012; Gopal et al. 2019).

## 2 Objective

Studies in RA patients receiving fatty acids or vitamin D along with conventional treatment have produced inconsistent results relating to their beneficial effects making it necessary to synthesize data from several, available trials to present conclusive evidence on their efficacy. The objective of this work is to provide a comprehensive and current meta‐analysis on the effectiveness of fatty acids and vitamin D on clinical scores in RA patients with a view to support clinical decisions for their use as adjuvant therapy for RA.

## 3 Methods

### 3.1 Search Strategy

A systematic literature search was conducted of MEDLINE (PubMed) and Cochrane Register of Controlled Trials (CENTRAL) in January 2025 from 2000 to 2025. The following search terms were used in various combinations: rheumatoid arthritis, fish oils, omega‐3 fatty acids, polyunsaturated fatty acids, vitamin D, cholecalciferol, ergocalciferol, 25‐hydroxy vitamin D, 24‐hydroxy vitamin D3, and calcifediol. Additionally, a comprehensive list of search terms including Medical Subject Headings (MeSH) terms were applied. The titles and abstracts of studies that were potentially relevant were scanned and the full text versions of the appropriate articles were read. Additional studies were identified by cross checking the reference lists of the relevant studies.

### 3.2 Study Selection or Inclusion/Exclusion Criteria

We included randomized controlled studies (RCT) that compared patients receiving fatty acids or vitamin D supplementation versus placebo, active controls (DMARD, MTX, corticosteroids, or conventional anti‐rheumatic drug treatment). Adult patients (≥ 18 years) with different phases with rheumatoid arthritis (RA) as defined by the American College of Rheumatology (ACR) or American Rheumatism Association (ARA) were included in our analysis. Patients were included if they were on stable DMARD, MTX, or glucocorticoid treatment. Non‐randomized studies, observational or retrospective studies, and those in pediatric populations were not included. Only studies published in English language were included in this study.

### 3.3 Data Extraction and Quality Assessment

Following identification of articles that met the inclusion criteria, data was extracted using a predefined data extraction form that included the following items: author name, year, patient characteristics (gender, age RA definition, concurrent medications), intervention characteristics such as dose, control information (placebo/active control), clinical outcome information, and duration of treatment and end point of the studies. One reviewer checked the articles and extracted the required information.

### 3.4 Risk of Bias Assessment

The Cochrane Collaboration's risk of bias tool was used to assess the methodological quality of the included studies. This tool includes the following criteria: randomization, allocation concealment, blinding, and completeness of follow‐up. The risk of bias for each item was graded as high, low, or unclear risk (Higgins et al. 2003).

### 3.5 Quantitative Data Synthesis

Meta‐analysis and statistical calculations were performed using Review Manager (RevMan, Version 5. Copenhagen: The Nordic Cochrane Center, The Cochrane Collaboration. 2020). Continuous outcomes such as clinical outcomes (DAS28, TJC, SJC, PGA, HAQ, and VAS) were expressed as the difference between the trial‐specific outcome value and baseline value and reported as means ± standard deviation (SD). The mean difference with associated 95% confidence intervals was calculated using the random‐effects model (inverse variance method). The random‐effects model was used to account for differences between the studies in terms of study design, participant, and intervention characteristics. Heterogeneity in the included studies was evaluated using *I* ^2^ statistic, with small heterogeneity for *I* ^2^ values of < 25%, moderate heterogeneity for *I* ^2^ values of 25% to 50%, and high heterogeneity for *I* ^2^ values > 50% (Higgins et al. 2003). Forest plots were constructed, and *p* < 0.05 was statistically significant. For each intervention type (fatty acid or vitamin D), each category of clinical outcome was subgrouped.

Publication bias was assessed by a funnel plot in which the standard error for each study was plotted against the mean difference for the DAS28 outcome.

## 4 Results

### 4.1 Identification of Studies

**FIGURE 1:**
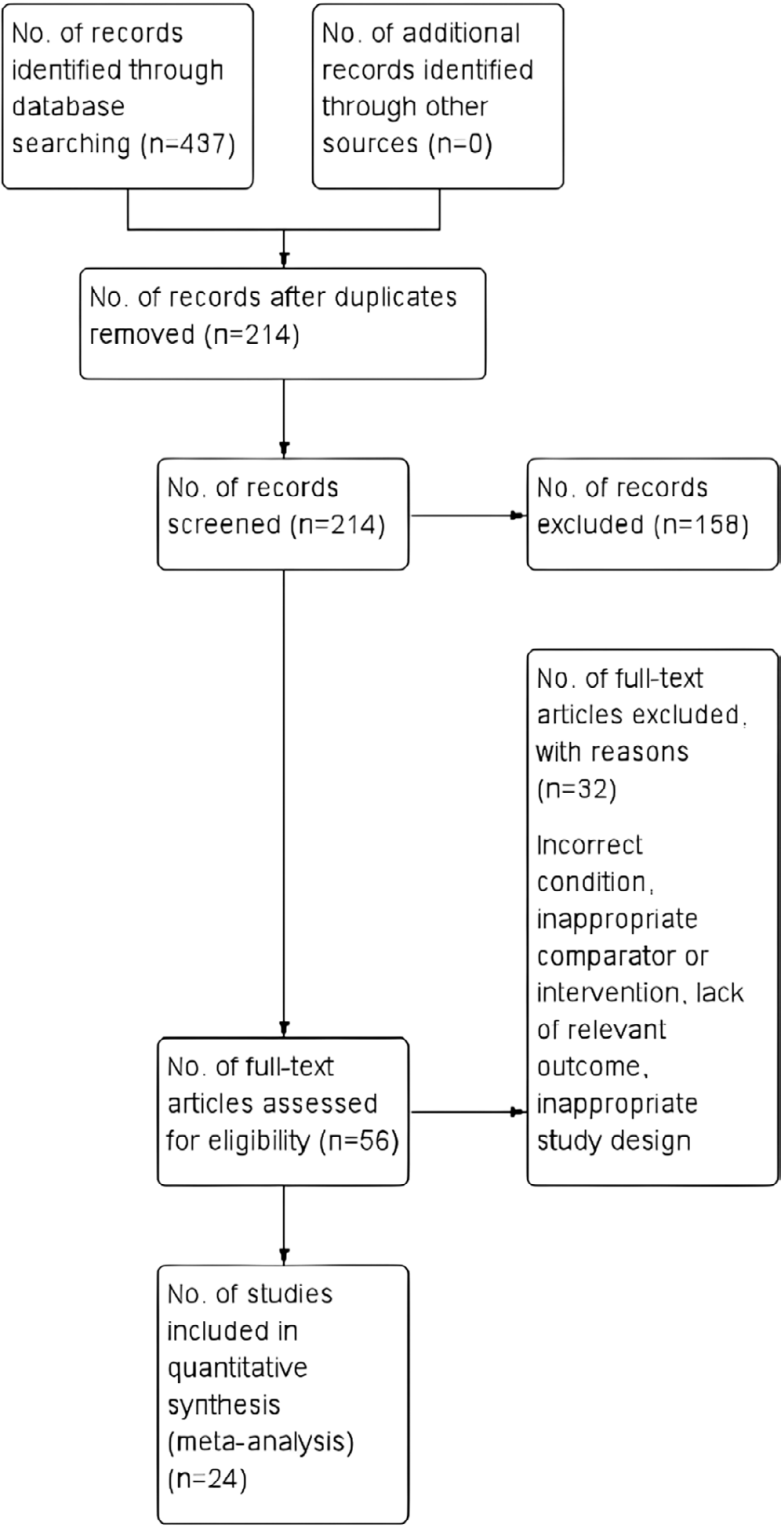
Flow chart for identification and inclusion of studies in the meta‐analysis according to preferred reporting items for systematic and meta‐analyses (PRISMA).

A total of 437 records were identified by database searching, of which 214 were screened by title and abstract. Duplicates and irrelevant records were removed (*n* = 158) and 56 RCTs were assessed for eligibility. However, 32 RCTs were excluded due to reasons such as inappropriate comparator, intervention, condition, population, lack of required outcome, and duplicate data. The process of selection is shown in Figure 1.

### 4.2 Study Characteristics

In total, 24 RCTs totalling were included in the meta‐analysis. Of these studies, 14 studies had fatty acids as the intervention group, and 10 reported the use of vitamin D supplementation as the intervention. In the fatty acid intervention group, 725 participants met the inclusion and for the vitamin D group, 988 participants met the inclusion criteria. These RCTs compared the use of fatty acids in the form of fish oil capsules, liquid nutritional supplements (these supplements that contain fish oil and vitamin D3), n‐3 PUFA capsules, cod liver oil capsules, or omega‐3 fatty acid capsules with either conventional RA treatment or placebo. Vitamin D as cholecalciferol, 1,25‐dihydroxy vitamin D, calcitriol, vitamin D2, were compared with patients receiving MTX, conventional RA treatment, or placebo. Table 1 shows the details of the interventions and controls and length of follow‐up of the included studies.

**TABLE 1:** Characteristics of the studies included in the meta‐analysis.

Study name	Patient characteristics	Intervention	Control	Follow‐up
Aryaeian et al. (2008)	Male and female patients, 19–69 years, RA per ACR guidelines	CLA capsule	Placebo	12 weeks
Bahadori et al. (2010)	Male and female patients, mean age 58 years, RA as per ARA, stable MTX and DMARD trt	Fish oil (EPA, DHA) IV	IV saline	22 weeks
Bansal et al. (2002)	Male and female patients, 18–60 years, RA as per ACR criteria	Vitamin D (400 IU 2x daily for 6 months) + MTX trt	MTX + placebo	6 months
Berbert et al. (2015)	Male and female patients, mean age 51 years, RA as per ACR criteria, NSAIDS, corticosteroids trt	Fish oil (omega‐3 fatty acid) capsule	Placebo (soy oil) capsule	24 months
Buondonno et al. (2017)	Female patients, early RA as per ACR criteria	MTX + GC + cholecalciferol (300,000 IU)	MTX + GC + placebo	3 months
Chandrashekara and Patted (2017)	Male and female patients, mean age 48 years, RA as per ACR criteria, stable DMARD trt	Vitamin D (60,000 IU/week) for 6 weeks 60,000 IU/month for 6 months	NA	NA
Das Gupta et al. (2009)	Male and female patients, RA as per ACR criteria	Intomethacin + omega‐3 fatty acid capsule	Indomethacin capsule	12 weeks
Dawczynski et al. (2009)	Male and female patients, RA as per ACR criteria, stable NSAIDs and corticosteroids trt	Fish and rapeseed oil (DHA, alpha LA, EPA)	Commercial dairy fat placebo	12 weeks
Dawczynski et al. (2018)	Male and female patients, RA as per ARA criteria, stable NSAID and DMARD trt	Diet with microalgae oil (DHA)	Diet with sunflower oil	10 weeks
Elfituri (2024)	Male and female patients, > 18 years, RA as per ACR criteria	DMARD+cholecalciferol (50,000 IU/week)	DMARD trt	12 weeks
Galarraga et al. (2008)	Male and female patients, mean age 59 years, RA as per ARA criteria, stable RA and NSAID trt	Cod liver oil (DHA, EPA) capsules	Placebo capsules	36 weeks
Gopinath and Danda (2011)	Male and female patients, RA as per ACR criteria	Calcium carbonate+1,25‐dihydroxy vitamin D (500 IU) + conventional trt	Calcium carbonate+conventional trt	NA
Hansen et al. (2014)	Male and female patients, RA	Vitamin D2 (50,000 IU 3×/week for 4 weeks)	Placebo	NA
Kolahi et al. (2010)	Female patient, RA as per ARA criteria	Fish oil (DHA, EPA) capsule + conventional trt	Conventional trt	3 months
Kumar et al. (2008)	Male and female patients, RA as per ACR criteria, DMARD trt	Starflower oil (GLA) capsule	Placebo (peanut oil) capsule	36 weeks
Li et al. (2018)	Male and female patients, > 18 years, RA as per ACR criteria	22‐oxa‐calcitriol (vit D3 analogue)	Placebo lactose powder	6 weeks
Park et al. (2013)	Male and female patients, mean age 48 years, RA as per ACR criteria, stable NSAID, DMARD and corticosteroid trt	n‐3 PUFA (EPA, DHA) capsules	Placebo (sunflower oil) capsules	16 weeks
Reed et al. (2014)	Male and female patients, 18–85 years, RA as per ARA criteria, stable DMARD or biologic trt	Fish oil (EPA, DHA) capsule	Borage seed capsule	18 months
Remans et al. (2004)	Male and female patients, active RA, stable DMARD and corticosteroid trt	Liquid nutritional supplement PUFA	Placebo	4 months
Salesi and Farajzadegan (2013)	Male and female patients, > 18 years, stable MTX trt	Vitamin D (50,000 IU/week)	Placebo	12 weeks
Soubrier et al. (2018)	Male and female patients, > 18 years, RA as per ACR criteria, stable DMARD and corticosteroid trt	Vitamin D (100,000 IU)	Placebo	24 weeks
Sundrarjun et al. (2004)	Male and female patients, mean age 46 years, stable DMARD trt	Fish oil (EPA, DHA) capsules	Placebo	12 weeks
Tyagi et al. (2024)	Male and female patients, 18–75 years, RA as per ACR criteria	Vitamin D (60,000 IU weekly) + DMARD	DMARD +placebo	3 months
Veselinovic et al. (2017)	Female patients, mean age 63 years, RA as per ACR criteria, stable DMARD, NSAID and corticosteroid trt	Fish oil (EPA, DHA) capsules	Conventional trt	12 weeks

Abbreviations: ACR, American College of Rheumatology; ARA, American Rheumatism Association; CLA, conjugated linoleic acid; DHA, docosahexaenoic acid; DMARD, disease‐modifying antirheumatic drugs; EPA, eicosapentaenoic acid; GC, glucocorticoids; GLA, gamma‐linolenic acid; MTX, methotrexate; NSAID, non‐steroidal anti‐inflammatory drug; PUFA, polyunsaturated fatty acid; RA, rheumatoid arthritis.

### 4.3 Characteristics of Participants

Studies were conducted in patients with RA as defined by American College of rheumatology (ACR) or American Rheumatism Association (ARA) criteria. Male and female patients, adults (> 18 years) and those receiving stable DMARD, MTX, or corticosteroid therapy were included in the analysis. Table 1 shows the patient characteristics.

### 4.4 Bias Assessment

**FIGURE 2:**
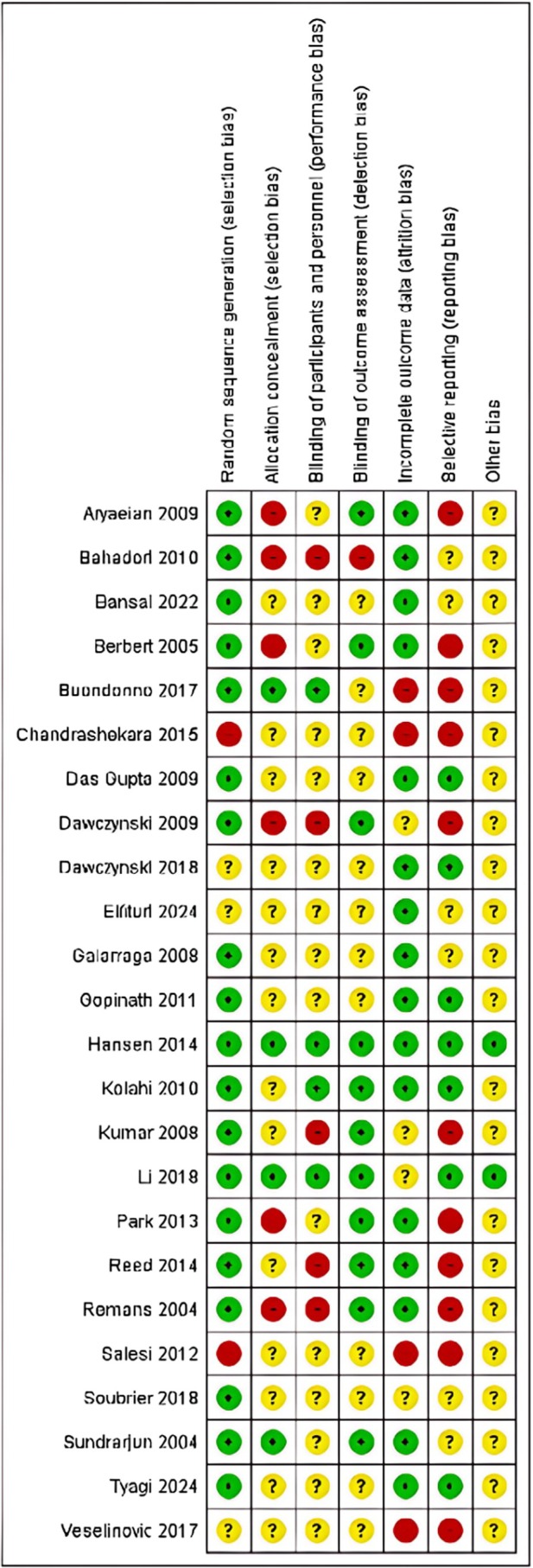
Risk of bias graph as per Cochrane Collaboration tool for studies included in the meta‐analysis (*n* = 24).

**FIGURE 3:**
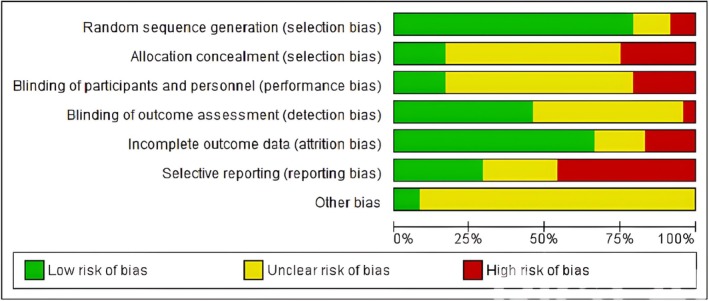
Risk of bias summary as per Cochrane Collaboration tool for studies included in the meta‐analysis (*n* = 24).

The results of the risk of bias evaluation are shown in Figure 2. Overall, there was an unclear risk of bias in the domain of allocation concealment, blinding of participants and personnel, and selective reporting necessitating caution in the interpretation of the results. Figure 3 is a risk of bias summary for the included studies. There was a high risk of bias in the selective reporting and allocation concealment domains.

**FIGURE 4:**
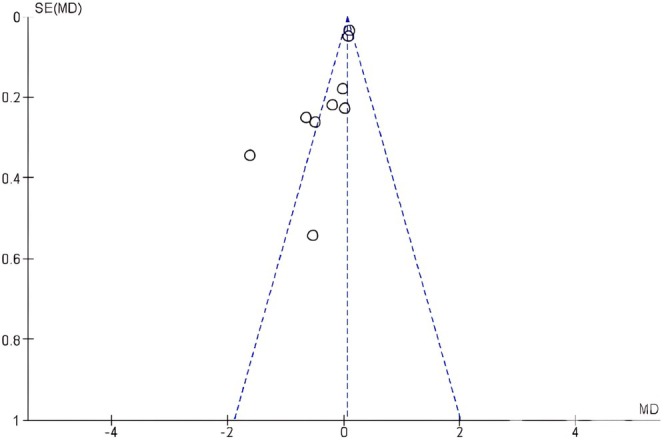
Funnel plot to assess publication bias in studies with fatty acid interventions, outcome: DAS28 scores.

**FIGURE 5:**
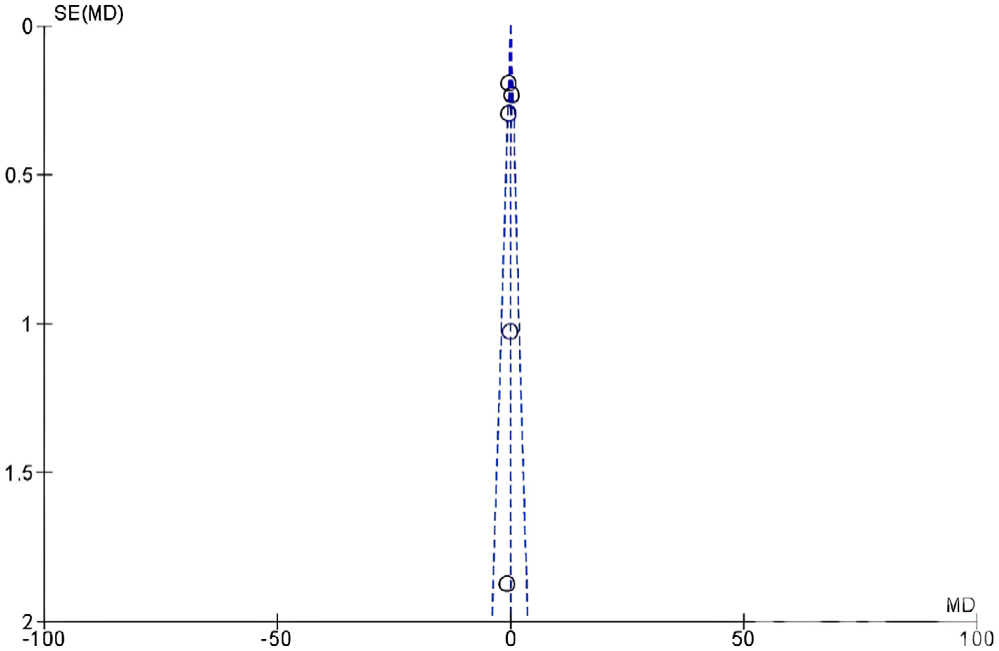
Funnel plot to assess publication bias in studies with vitamin D interventions, outcome: DAS28 scores.

The funnel plot was not symmetrical for the DAS28 outcome for both studies involving either fatty acids or vitamin D interventions indicating the possibility of publication bias (Figures 4 and 5).

### 4.5 Meta‐Analysis Results

#### 4.5.1 DAS28 Outcome

**FIGURE 6:**
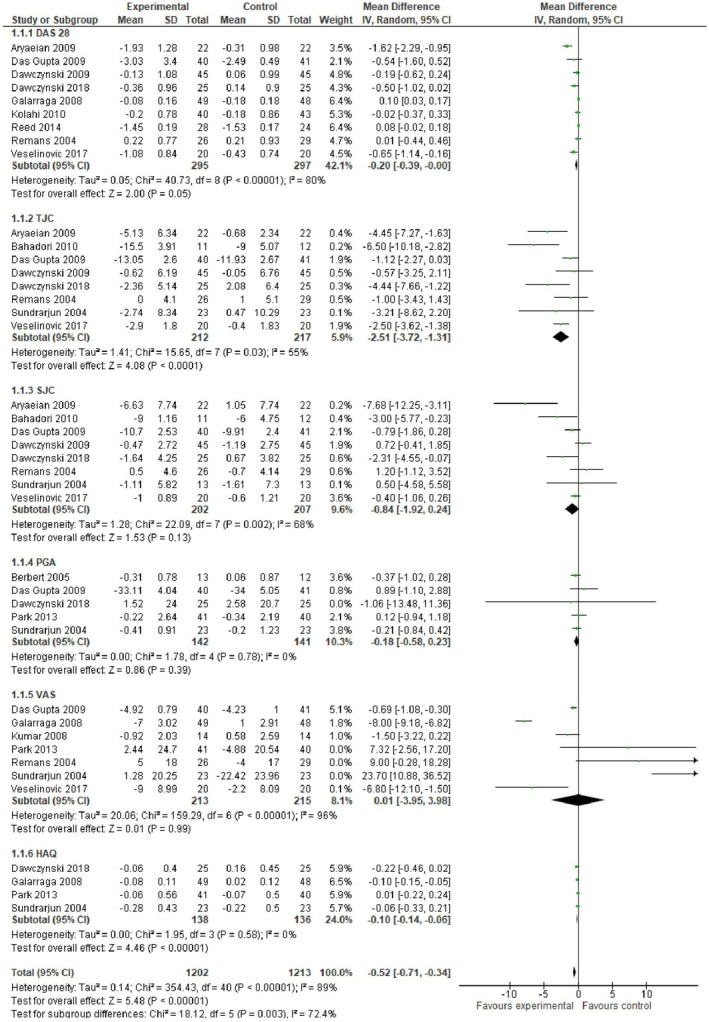
Forest plot for clinical scores following fatty acid intervention in rheumatoid arthritis patients. Values represent mean difference and 95% CI. Experimental: Fatty acids; control: Placebo, conventional treatments.

**FIGURE 7:**
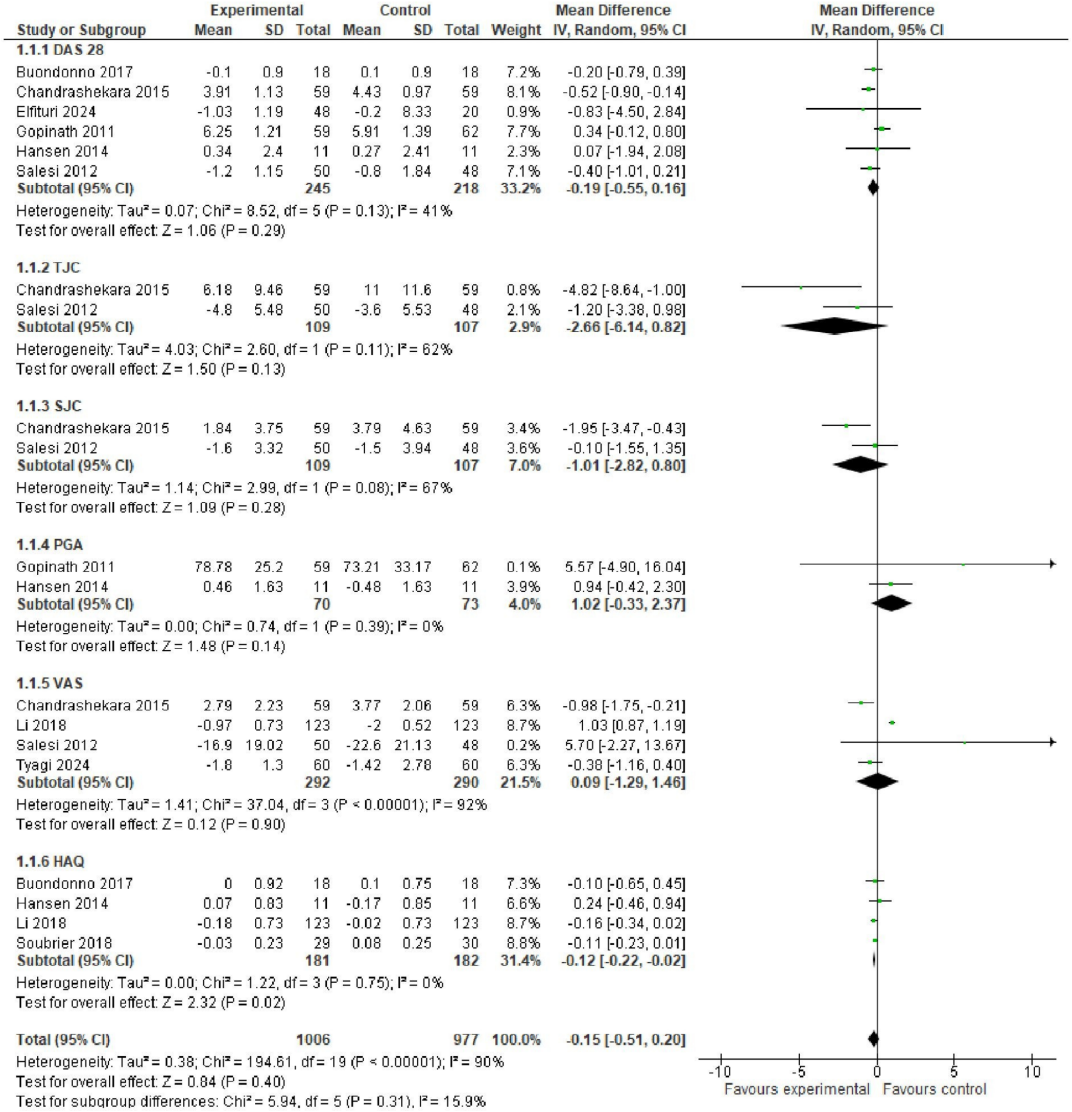
Forest plot for clinical scores following vitamin D interventions in rheumatoid arthritis patients. Values represent mean difference and 95% CI. Experimental: Vitamin D; control: Placebo, conventional treatments.

The disease activity score in 28 joints outcome was determined from each study at the baseline and end of follow‐up and the mean of the difference and standard deviation was determined for the intervention and control groups for each study. Figures 6 and 7 show the DAS28 outcomes determined as the weighted mean difference of these differences by meta‐analysis using the random‐effects inverse variance model. The mean differences and 95% confidence intervals are shown. There was a significant decrease in the DAS28 score in patients receiving fatty acid supplementation relative to controls (greater decrease implies a significant improvement in RA) (MD −0.20, −0.39 to 0.00, *p* = 0.05, *I* ^2^ = 80%) (*n* = 9 studies) (Figure). However, the DAS28 improvement was not significant in patients receiving vitamin D (MD −0.19, −0.55 to 0.16, *p* = 0.29, *I* ^2^ = 41%) (*n* = 6 studies) (Figure 6).

#### 4.5.2 Tender Joint Count (TJC)

TJC was evaluated at the beginning and end of each study and the difference of the means at the two time points and standard deviation was determined for the studies utilizing fatty acids (*n* = 8 studies) and those with vitamin D supplementation (*n* = 2 studies) (Figures 6 and 7, respectively). There was a significant decrease in TJC score in patients receiving fatty acids (MD −2.51, −3.72 to −1.31, *p* < 0.0001, *I* ^2^ = 55%) (Figure 5) whereas the TJC did not significantly improve following vitamin D supplementation (MD −2.66, −6.14 to 0.82, *p* = 0.13, *I* ^2^ = 62%) (Figure 6).

#### 4.5.3 Swollen Joint Count (SJC)

The results for the differences in SJC in patients receiving fatty acids and vitamin D are shown in Figures 5 and 6. There were no significant differences in the mean differences of SJC between either intervention group, fatty acids, or vitamin D, compared to controls. For the fatty acid group (*n* = 8 studies), MD −0.84, −1.92 to 0.4, *p* = 0.13, *I* ^2^ = 68% (Figure 6) and for the vitamin D group (*n* = 2 studies), MD −1.01, −2.82 to 0.80, *p* = 0.28, *I* ^2^ = 67% (Figure 7).

#### 4.5.4 Patient Global Assessment (PGA)

The results for the differences in PGA scores at baseline and end of the study and meta‐analyzed mean differences for the fatty acid and vitamin D groups are shown in Figures 6 and 7, respectively. In both cases, there was no significant improvement in PGA following treatment with either fatty acids or vitamin D. The mean difference and 95% CI for the PGA score in the fatty acids group is MD −0.18, −0.58 to 0.23, *p* = 0.39, *I* ^2^ = 0% (*n* = 5 studies), and for the vitamin D group it was.

MD1.02, −0.33 to 2.37, *p* = 0.14, *I* ^2^ = 0% (*n* = 2 studies).

#### 4.5.5 Visual Analogue Scale (VAS)

The VAS scores for the fatty acid and vitamin D treatments are shown in Figures 6 and 7, respectively. There was no significant difference in the VAS score in the fatty acid group MD 0.01, −3.95 to 3.98, *p* = 0.99, *I* ^2^ = 96% (*n* = 7 studies), or in the vitamin D group MD 0.09, −1.29 to 1.46, *p* = 0.90, *I* ^2^ = 92% (*n* = 4 studies).

#### 4.5.6 Health Assessment Questionnaire (HAQ)

In the fatty acid and vitamin D groups, the HAQ scores were significantly lower, showing an improvement when compared to the controls. Figure 6 shows the HAQ score for the fatty acid group (*n* = 4 studies) as MD −0.10, −0.14 to −0.06, *p* < 0.00001, *I* ^2^ = 0%, and Figure 6 shows the HAQ score for the vitamin D group as MD −0.12, −0.22 to −0.02, *p* = 0.02, *I* ^2^ = 0% (*n* = 4 studies).

The moderate to high heterogeneity between studies for most outcomes can be attributed to differences in patient characteristics, intervention types and doses, length of follow‐up, and subjective assessment of clinical outcomes such as DA28 that include physician and patients' assessments that may not necessarily be consistent.

## 5 Discussion

This paper is a comprehensive and updated synthesis on evidence for the use fatty acids and vitamin D supplementation in RA patients. Important clinical outcomes instruments such as DAS28, TJC, SJC, PGA, VAS, and HAQ were used to determine the efficacy of these interventions (Table 2). These instruments capture different facets of RA severity and include different constructs such as disease symptoms, physical function, pain, and quality of life and are often used in conjunction with laboratory measures such as erythrocyte sedimentation rate (ESR), C‐reactive protein (CRP), rheumatoid factors (RF), and anti‐cyclic citrullinated peptide (anti‐CCP) antibodies that are used as markers of inflammation and risks of RA. In the present meta‐analysis, control groups were either placebo or conventional RA therapies such as DMARDs, and corticosteroids and patients were diagnosed with RA using ACR or ARA criteria.

**TABLE 2:** Clinical outcomes reported in the included studies.

Study name	DAS 28	TJC	SJC	PGA	VAS	HAQ	ACR
Aryaeian et al. (2008)	✓	✓	✓				
Bahadori et al. (2010)		✓	✓				
Bansal et al. (2002)							✓
Berbert et al. (2015)				✓			
Buondonno et al. (2017)	✓					✓	
Chandrashekara and Patted (2017)	✓	✓	✓		✓		
Das Gupta et al. (2009)	✓	✓	✓	✓	✓		
Dawczynski et al. (2009)	✓	✓	✓				
Dawczynski et al. (2018)	✓	✓	✓				
Elfituri (2024)	✓						
Galarraga et al. (2008)	✓				✓	✓	
Gopinath and Danda (2011)	✓			✓			
Hansen et al. (2014)	✓			✓		✓	
Kolahi et al. (2010)	✓						
Kumar et al. (2008)					✓		
Li et al. (2018)					✓	✓	
Park et al. (2013)				✓	✓	✓	
Reed et al. (2014)	✓						
Remans et al. (2004)	✓	✓	✓		✓		
Salesi and Farajzadegan (2013)	✓	✓	✓		✓		
Soubrier et al. (2018)						✓	
Sundrarjun et al. (2004)		✓	✓	✓	✓	✓	
Tyagi et al. (2024)					✓		
Veselinovic et al. (2017)	✓	✓	✓		✓		

Abbreviations: ACR, American College of Rheumatology score; DAS28, Disease Activity Score 28‐joint count; HAQ, Health assessment questionnaire; PGA, Patient global assessment; SJC, Swollen joint count; TJC, Tender joint count; VAS, Visual analogue scale.

Risk of bias assessments using the Cochrane tools showed a high risk to unclear risk of biases in the domains of allocation concealment, blinding of participants and personnel, and detection bias. Allocation concealment may be difficult when fish oil capsules containing fatty acids are given due to distinct odor which may be difficult to mask even if placebo capsules with identical appearance is administered. Blinding of participants is also an issue due to the distinct taste and side effects such as gastrointestinal disturbances which are often caused by fish oils. Similar challenges also occur with vitamin D regarding side effects and vitamin D levels assessment that can compromise blinding. The high heterogeneity (*I* ^2^ value) in the subgroups could be attributed to differences in patient populations (stage and severity of RA, concomitant medications such as DMARDs and steroids, level of physical activity, age), treatments (type of fatty acid or vitamin D and dose), and interventions (conventional treatment or placebo) of the trials included together.

For the meta‐analysis evaluating the effects of fatty acid supplementation, 14 RCTs were included. The DAS28 scores, TJC, and HAQ outcomes were significantly improved with fatty acid supplementation however, the HAQ scores differences were not statistically significant between the groups. The DAS outcome although borderline statistically significant (*p* = 0.05) may not be clinically relevant as several studies were not statistically significant. Although, the SJC and PGA assessments were not statistically significant they could be clinically significant. The DAS28 score includes physical and psychological domains and is based on parameters such as TJC, SJC, PGA, and ESR/CRP. The improvement in DAS score could be due to significant improvement in the individual parameters such as TJC, SJC, and PGA scores in this composite assessment tool in the fatty acid group. Omega‐3 fatty acids such as eicosapentaenoic acid (EPA) and docosahexenoic acid (DHA) are known to modulate the inflammatory responses by inhibiting the arachidonic and proinflammatory pathway thereby decreasing the production of cytokines, IL‐1β, IL6, and TNF‐α that cause inflammation (Veselinovic et al. 2017; Lee et al. 2012). These beneficial effects on inflammation were shown to decrease TJC, SJC, and improve physical function and patients' overall health. However, there was no significant improvement in VAS score that relates to pain indicating that not only inflammation, but other psychosocial factors may be involved in the pain stimuli. Previous meta‐analyses on fatty acids supplements showed no significant improvement in TJC, SJC, and physical function though they were marginally better than the control group but included studies using only high dose‐PUFAs (Lee et al. 2012). Other meta‐analyses have shown results that conflicting but mainly a beneficial effect on TJC and morning stiffness but a non‐significant benefit on SJC and PGA (Fortin et al. 1995; Goldberg and Katz 2007). The most recent meta‐analyses by Turk et al. showed a non‐significant improvement in TJC, SJC, pain, and PGA whereas DAS28 was significantly improved in the group receiving fatty acid interventions.

However, in the case of the vitamin D trials, although the HAQ showed a significant improvement and DAS28 could be clinically relevant although not statistically significant, all other outcomes were not significantly better in the vitamin D groups compared to placebo/active controls. There were only two studies reporting SJC, TJC, and PGA outcomes, limiting the use of this data to make a definitive conclusion. The improvement in DAS28 could have been attributed to improvements in clinical markers such as ESR and CRP, which are also included in the DAS28 construct. Furthermore, differences in the types of vitamin D forms such as cholecalciferol, 1,25‐dihydroxy vitamin D, 22‐oxacalcitriol, and doses and duration of treatment can affect their efficacy in rheumatoid arthritis, resulting in heterogeneous results. There is no strong evidence of the role of vitamin D in immune responses and inflammatory processes, preventing a conclusive basis for its administration in RA patients. Earlier meta‐analyses have reported an effect of vitamin D doses and duration on clinical outcomes such as TJC and VAS, but because of the limited number of trials available, subgroup analyses were not performed as this would affect the statistical significance (Turk et al. 2023; Guan et al. 2020; Al‐Saoodi et al. 2024).

## 6 Limitations of the Study

Although the current study involves a comprehensive quantitative view on the beneficial effects associated with the use of fatty acids and vitamin D in rheumatoid arthritis patients, it has certain shortcomings that must be considered prior to interpretation of results and use of these therapies in clinical settings.

Firstly, the meta‐analysis only included studies that were published in English and for which full‐text articles were available from peer‐reviewed journals. Due to this, the available number of trials related to the use of these non‐pharmacological therapies was limited, particularly for vitamin D trials, which can produce results that may not be statistically robust. Furthermore, the number of subjects in the trials is also < 50 for most trials, which affects the statistical power of the study. The dosages and types of supplements differ between the studies, contributing to heterogeneity, which also casts doubt on the meta‐analysis results. However, separating the trials into homogenous groups does not produce enough subjects to make a valid conclusion, so that trials with heterogenous interventions, controls, and patient characteristics must be pooled together. The background medication that the patients were receiving also differs significantly, and this can affect the outcomes. The length of follow‐up between the trials varied and was also not adequate in some cases (≤ 3 months) making it difficult to capture the actual efficacy of the therapies. Overall, standardized outcome measures and longer follow‐up durations are necessary to capture all outcomes. The effectiveness of these interventions is also dependent on the stage and severity of RA, which was not reported in all the studies, leading to inconclusive evidence to support the use of fatty acids or vitamin D in certain subgroups of RA patients. Other confounding variables, such as baseline levels of vitamin D, physical activity, co‐interventions, diet, and fatty acid intake were not considered while pooling the results. In addition, since the outcomes used were clinical outcomes and are both physician‐assessed and patient‐reported, it is possible to have discrepancies between physician assessment of the disease state and patients' assessment, which may be based on psychological benefits and more abstract concepts. Inter‐rater variability amongst physicians and adequate training and education for patients on understanding these questionnaires are necessary so that the outcomes are accurate and reliable. Additionally, correlation of these outcomes with objective clinical markers such as CRP levels and ESR values will further strengthen the results.

## 7 Conclusion

The findings of this meta‐analysis demonstrate the benefits of fatty acids and vitamin D as complementary non‐pharmacological interventions that can be used to assist in decreasing pain, improving patients' quality of life, and reducing symptoms of arthritis. Fatty acids were shown to improve outcomes related to inflammation such as tenderness and swelling in the joints and contribute positively to the functional capacity of patients. The effectiveness of vitamin D supplementation was moderate with respect to outcome measures. However, this meta‐analysis included a limited number of trials with heterogeneity and methodological limitations requiring the need for larger, multi‐centre trials with homogenous patient groups and interventions with longer follow‐up durations to produce robust evidence.

## Author Contributions

**Bing Xu:** conceptualization (equal), data curation (equal), formal analysis (equal), investigation (equal), methodology (equal), writing – review and editing (equal). **Dongdong Liang:** conceptualization, investigation, writing – review and editing. **Guangfeng Chen:** data curation (equal), formal analysis (equal), investigation (equal), methodology (equal), writing – original draft (equal).

## Ethics Statement

The authors have nothing to report.

## Consent

The authors have nothing to report.

## Conflicts of Interest

The authors declare no conflicts of interest.

## Data Availability

Upon reasonable request, the corresponding author will provide access to the requested information.
